# Effects of drying eggs and egg storage on hatchability and development of *Anopheles arabiensis*

**DOI:** 10.1186/1475-2875-12-318

**Published:** 2013-09-12

**Authors:** Inamullah Khan, David Damiens, Sharon M Soliban, Jeremie RL Gilles

**Affiliations:** 1 Nuclear Institute for Food and Agriculture, G.T. Road, Peshawar, Pakistan; 2 Insect Pest Control Laboratory, Joint FAO/IAEA Division of Nuclear Techniques in Food and Agriculture, International Atomic Energy Agency, Vienna, Austria

**Keywords:** Sterile insect technique, Anopheles, Mass rearing, Egg management, Volumetric estimation

## Abstract

**Background:**

The mass rearing of insects requires a large colony from which individuals can be harvested for sterilization and release. Attention is given to larval food requirements and to handling and rearing conditions to ensure predictability and synchrony of development. Maximizing production requires optimized adult holding to ensure mating success, blood feeding and oviposition. Appropriate egg storage and harvesting is necessary to compensate any unpredicted reduction in egg production.

**Methods:**

*Anopheles arabiensis* eggs were collected on wet filter paper in eggs cups. The eggs were cleaned and then dried over a suction device with adjustable speed and time. The effects of drying, storage time and storage condition (wet, dry and bulk with relative humidity 75 ± 5% and storage temperatures of 10, 15 and 20°C) on hatch rate, duration of larval stages (L1 to pupal stage), duration of L1 to adult emergence, survival of L1 to pupal stage and the survival of L1 to adult emergence were investigated. Post drying and post storage hatch rates were determined by counting hatched and unhatched eggs and were confirmed by counting the viable larvae in the rearing medium.

**Results:**

The hatch rate of eggs dried at wind speeds of 1.0 or 1.8 m/s was not significantly different from the control, but eggs dried at 3.0 m/s resulted in very low (64%) hatchability as compared to the control (82%). Eggs stored at 20°C and 75 ± 5% RH in bulk in an aerated vial showed better survival than eggs stored in wet or dry conditions at 10 or 15°C. No significant changes in larval duration and survival were recorded after six days of bulk storage.

**Conclusion:**

*Anopheles arabiensis* eggs can be stored in bulk at 20°C and 75 ± 5% RH for six days without any decrease in hatch rate, and up to 9 days with no impact on larval development.

## Background

The sterile insect technique (SIT) is a species-specific and environmentally friendly method of insect pest control based on the release of large numbers of sterile insects [1]. Mass-reared (male) insects exposed to ionizing radiation prior to release transfer their sterile sperm to wild females during mating, causing a reduction in the fertility of the female target population resulting in a progressive decline of the pest population. The efficacy of the SIT relies on maintaining a continuously high ratio of sterile to fertile males within the target area and the competitiveness of these sterile males. For example, from 1977 to 1979 on the Pacific coast of El Salvador, 0.5 to 1.25 million sterile male pupae were released daily during the SIT programme against *Anopheles albimanus*[2,3].

The Tropical Medicine Research Institute in Sudan, with the support of the Food and Agriculture Organization of the United Nations (FAO) and the International Atomic Energy Agency (IAEA) has initiated a study to assess the feasibility of integrating the SIT for the area wide control of the important malaria vector *Anopheles arabiensis* along the Nile River in Northern State, Sudan. To control *An. arabiensis* in Sudan, the production of one million *An. arabiensis* sterile males per day is anticipated [4]. To achieve this goal and obtain a sustainable and affordable production, the Insect Pest Control Laboratory (IPCL) of the Joint FAO/IAEA Division of Nuclear Techniques in Food and Agriculture has been supporting this project with the development of low cost diets [5,6], mass-rearing equipment and optimized rearing protocols [7]. A larval rearing unit composed of a rack containing 50 trays, a larvae-pupae separator [8] and an adult mass-rearing cage [9] have been developed and evaluated for *An. arabiensis* and are now being transferred to field projects for further validation under operational conditions.

A crucial aspect in the rearing of mosquitoes, such as *An. arabiensis*, is the management of the large quantity of eggs produced in the mass-rearing cage. Handling and measuring high volumes of fresh eggs is tedious and impractical, primarily due to the sticky properties of fresh eggs. Moreover, *Anopheles* eggs remain viable for a short time only on wet substrates [10] and efforts must be directed towards determining the drying and storing conditions for *Anopheles* eggs that would be least detrimental to development parameters. Therefore, methods of drying eggs have been developed to allow manipulation and measurement in the context of the SIT, for instance for *An. albimanus*, *Anopheles quadrimaculatus* and *Anopheles stephensi*[11-14]. Eggs of *An. albimanus* and *An. quadrimaculatus* can survive the drying process and withstand considerable periods of storage [15]. After drying, eggs could be easily manipulated and the number of eggs estimated volumetrically. Moreover, due to the high number of eggs and adults that must be produced per day, storage of eggs could help manage insect production objectives.

The present study was conducted to determine the effects of drying, storage temperature, storage conditions (dry, wet, bulk) and storage time on the hatchability of *An. arabiensis* eggs and the post-hatch life parameters including larval duration and survival to adult emergence.

## Methods

### Egg collection and cleaning

The larval stages of *An. arabiensis* Dongola strain were reared in 1 litre deionized water held in plastic trays (30 × 40 × 8 cm). Larvae were fed a diet of ground fish food (Koi Floating Blend®, Aquaricare®, New York, USA). Pupae were separated and put into metal-framed cages (each 60 × 60 × 60 cm) (BugDorm®). Adults had constant access to 10% sucrose plus 0.2% methylparaben solution [5]. The rearing conditions were 27 ± 1°C and 60 ± 10% RH with a 12:12 LD photoperiod including dusk (1 h) and dawn (1 h). Each cage contained approximately 2000–3000 adults. For egg production females were given a blood meal (mechanically defibrinated bovine blood). Gravid females oviposited in plastic cups with black lining on the sides and a wet sponge on the bottom over which a filter paper disc was placed.

**Figure 1:**
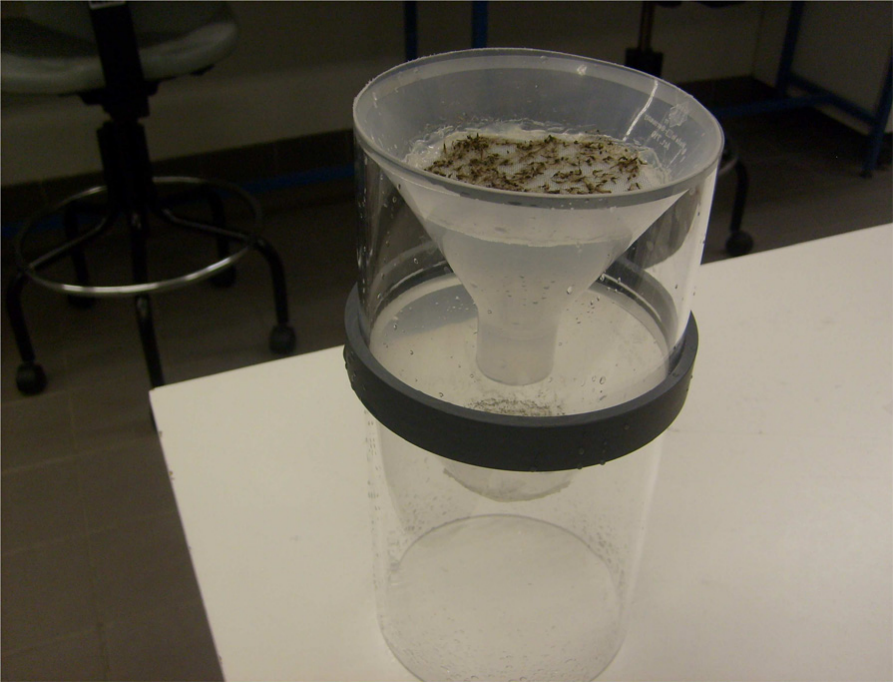
**Apparatus for cleaning and drying eggs.** Detachable funnels and rings holding the funnels above each other.

Eggs were collected and then cleaned with an apparatus (Figure 1) constructed from transparent Plexiglas pipes cut into two pieces, 9 and 12 cm long. Two plastic funnels (each 12 cm diameter) were used. A polyester 500-micron mesh was glued with silicon to the centre of one funnel. The second funnel was cut in the middle leaving a hole (6 cm diameter) at the bottom. Another polyester cloth (that retained eggs) was glued over the cut end of the second funnel. Both funnels were fitted over each other with the help of a ring. Eggs collected on the blotting paper in the egg cups were washed into the upper funnel with the help of a wash bottle. Excess debris and insect particles were collected in the top funnel but eggs could pass through it and were collected on the second mesh. Eggs were then washed for 1 min with a gentle flow of deionized water.

### Effect of egg drying on hatch rate

**Figure 2:**
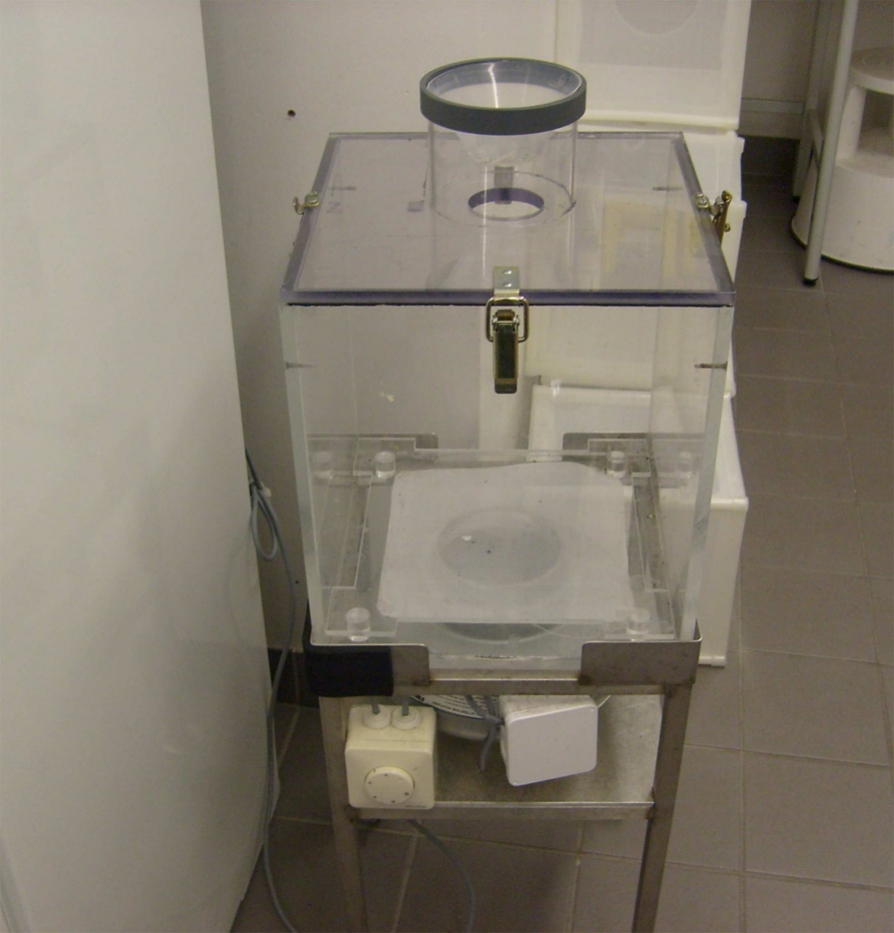
Suction device with adjustable fan speed for drying eggs.

The lower portion of the apparatus with the funnel and clean eggs was detached and placed over a suction device with adjustable speed (Figure 2). The wind speed and drying duration were adjusted so that all eggs dried and no clumping was apparent. Three wind speeds (1.0, 1.8 and 3.0 m/s) and four drying durations (10, 15, 20 and 25 min) were tested. Wind velocity was measured using a Testo 417 anemometer (Testo Ltd, Alton, UK). For each combination of wind speed and drying duration, three replicates of 200 eggs were used. At the end of each treatment, eggs were placed in cups lined on the inside with 2.5-cm-wide filter paper strips and containing 40 ml deionized water (providing 1 cm water at the bottoms of the cups). The tops of the cups were covered by placing a tray over them to reduce evaporation. Control eggs were taken directly from the filter paper in egg cups and divided into three replicates of 200 eggs as described above. All cups were placed in a temperature-controlled rearing room at 27°C for hatching.

Eggs sticking to the sides of the filter paper after 24 h were rinsed down into the water. Filter papers lining the sides of the hatching cups were removed with forceps after 48 h and dried on tissue paper. Hatched and unhatched eggs sticking to the sides of the filter papers were counted with a stereomicroscope. The hatch rate was confirmed by counting the number of viable larvae in each cup.

### Effects of egg storage on hatch rate

Based on the results of the experiment on wind speed (see results section), all eggs were dried at 1.8 m/s for 20 min. The following experiment was conducted to determine the most suitable temperature (10, 15 or 20°C), duration (1 to 8 days) and condition (dry, wet or bulk) for extended periods of storage. In the dry condition, eggs were stored in 200 μl Eppendorf tubes with caps closed; in the wet condition, eggs were stored in 200 μl Eppendorf tubes with wet cotton at the top end. For both of the conditions, 90 tubes with 200 eggs each were prepared, with 30 tubes being stored at each of the three temperatures. Relative humidity was held at 75% ± 5 in storage facilities.

The hatch rates of eggs stored in either wet or dry conditions were determined daily by withdrawing three tubes from each storage temperature and dropping eggs in cups containing 40 ml deionized water. In the bulk method about 10,000 dry eggs were stored in 1.5 ml graduated glass bottles with screw caps modified for air circulation with a 2-mm hole at the upper end. Each bottle with eggs was then stored at 10 or 15°C in an incubator or at 20 ± 2°C in a temperature-controlled room (75 ± 5% RH). The hatch rate of eggs stored in bulk was determined daily from approximately 600 eggs that were replicated in three hatching cups each with about 200 eggs. All cups with water and eggs were then placed in a clean tray and covered with another tray to prevent contamination and water evaporation. The tray with the cups was placed in a temperature-controlled rearing room at 27 ± 1°C, 60 ± 10% RH and a 12:12 LD photoperiod including dusk (1 h) and dawn (1 h). The status of egg hatch was observed with a stereomicroscope after 48 h by checking hatched and unhatched eggs and the number of larvae in the medium.

### Effects of egg storage duration on larval development parameters

Eggs dried at 1.8 m/s for 20 min were stored at 20 ± 2°C and 75 ± 5% RH in bulk as described above. The effects of storage duration on hatch rate, duration of larval stages (L1 to pupal stage), duration of L1 to adult emergence, survival of L1 to pupal stage and survival of L1 to adult emergence were observed. The hatch rate was determined by counting eggs on paper and counting L1 from three cups of 200 eggs as described above. For assessment of larval developmental parameters, 150 μl of 1% (w/v) fish food (Koi Floating Blend®) were added to the water in hatching cups as food for hatching larvae. To test the effects of storage duration on duration of larval stages (L1 to pupal stage), duration of L1 to adult emergence, survival of L1 to pupal stage and survival of L1 to the adult emergence, one subsample of 32 L1s from each replicate cup were transferred into standard 90-mm-diameter disposable polystyrene Petri dishes with 32 ml of deionized water. Larvae in each dish were fed with 640 μl of 1% (w/v) Koi fish food on a daily basis until pupation. Larval mortality was noted (based on dead larvae in the dishes). Once pupae had formed they were picked up with a plastic pipette and transferred to plastic tubes containing about 2 ml water until adult emergence.

### Volumetric estimation of the number of dried eggs

Dry eggs were poured into a 1.5 ml screw-capped graduated glass bottle, the cap of which was modified into a tube shape with a 2-mm hole at the top to permit the transfer of eggs into another tube. Eppendorf tubes were marked at different volumes: 10, 20, 30, 40 50,100 and 150 μl using automatic pipettes for the measurement, each replicated three times. For each volume, these tubes were filled to the mark with dried *An. arabiensis* eggs from the graduated glass bottles. All tubes were centrifuged for 2–3 sec so that the eggs reached the measured level. Once the tube was filled, the eggs were poured onto a gridded paper at the bottom of a Petri dish. Eggs were spread over the gridded area with a camel hair brush and counted with a stereomicroscope. The procedure was repeated three times resulting in three egg quantity estimations for each volume. Correlation between the number of eggs and volume was done using Pearson correlation software.

### Statistical analysis

Data on egg drying time and wind speed were analyzed using the two-way ANOVA followed by Tukey’s HSD test. To study the effect of storage period, temperature and storage condition on hatching rate, correlation between percent hatch and storage period for each temperature/storage condition were tested and compared. Pearson correlation coefficient were calculated and tested for each relationship. Least-Squares regression lines were then determined and slopes and intercepts of lines were tested with Analysis of variance in General Linear Model [16] using storage period as covariate. If the null hypothesis for ‘treatment temperature/storage condition’ was rejected (i.e. if its p-value is less than alpha), the intercepts of the regression models were not all equal. If the null hypothesis for ‘storage period * treatment temperature/storage condition’ was rejected, then the slopes of the regression models were not all equal. Pearson correlation coefficient calculation and tests of slopes and intercepts were performed using Minitab release 16 (Minitab, State College, PA). The effects of bulk storage at 20°C on hatch rate, duration of larval stages (L1 to pupal stage), duration of L1 to adult emergence, survival of L1 to pupal stage and survival of L1 to adult emergence were analyzed using ANOVA, with a completely randomized design, followed by Tukey’s HSD test. Pearson’s correlation coefficient between volume and the number of eggs were determined. Simple linear regression was performed between the number of eggs and volume. All statistical analyses were done using Statistix 8.1 (Analytical Software, Tallahassee, FL).

## Results

### Effect of egg drying on hatch rate

Effects of drying time and wind speed on percent hatch (± SD) of *An. arabiensis* eggs have been summarized in Table 1. Two way ANOVA shows extremely significant effect of both drying duration (F = 12.4, DF = 3, P < 0.001) and wind speed (F = 79.9, DF = 3, P < 0,001) on the egg hatch rate. High significant interaction between drying duration and wind speed has been also observed (F = 8.4, DF = 9, P < 0.001). The significant interaction means that the effect of one variable is significantly different for each levels of the other variable. Tukey post-tests indicate that there is no significant differences in hatch rate between 10, 15, 20 min drying duration. The drying duration of 25 minutes shows a significant difference with all the other times. No significant difference has been observed between controls, 1.0, and 1.8 m/s wind speed treatment groups, but the 3.0 m/s treatment group was significantly different from the others. Thus the only treatment where all eggs were dried at the end and that showed no significant effect of the drying duration and wind speed was a speed of 1.8 m/s for 20 min. This combination of wind speed and duration was used in the remaining experiments.

**Table 1:** **Effects of drying time and wind speed on percent hatch of *An. arabiensis *eggs**

**Wind speed (m/s)**	**Drying time (min)**			
	**10**	**15**	**20**	**25**
1.0	83.3 ± 4.3	81.0 ± 5.6	80.0 ±2.8	81.9 ±4.5
1.8	79.9 ±6.1	81.4 ± 5.9	**81.86 ±0.9**	**75.44 ±4.5**
3.0	67.5 ±1.6	**66.3 ±2.6**	**64.2 ±3.0**	**35.8 ± 8.0**
Control	81.7 ± 2.5	81.7 ±2.5	81.7 ±2.5	81.70 ± 2.5

The bold indicates that the eggs were totally dried following treatments.

Totally dried mean, eggs were dry and not clumping with each other.

### Effects of egg storage on hatchability

**Figure 3:**
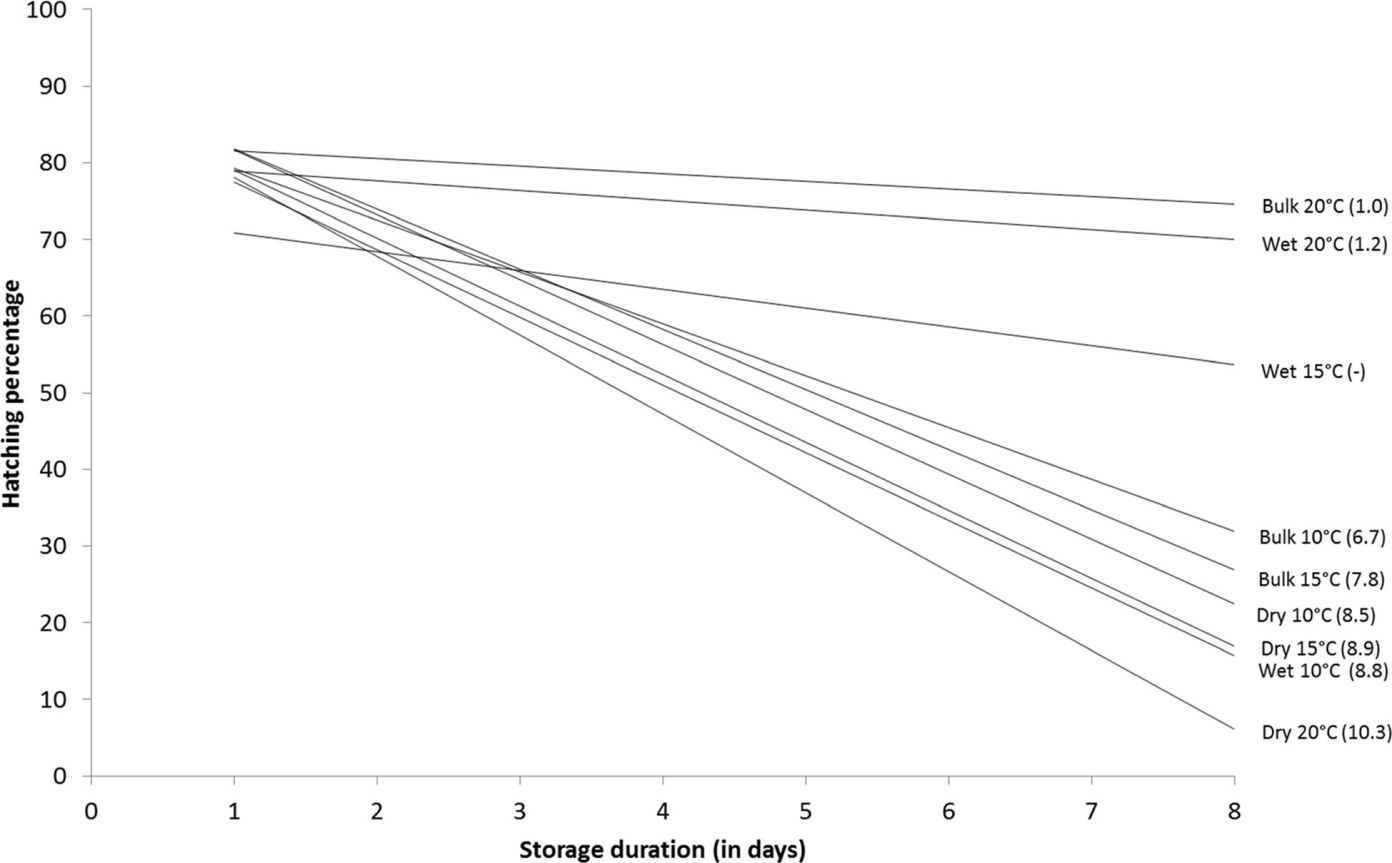
**Relationship between percent hatch and storage duration for each treatment temperature/storage condition.** Slope of each regression line is indicated in parentheses (no slope was given for Wet 15°C since the Pearson correlation coefficient was not significant).

Effects of storage period, storage condition and temperature on per cent hatch (± SD) of *An. arabiensis* eggs have been summarized in Table 2. To study the effect of the different treatments on hatching rate, relationship between percent hatch and storage period for each treatment temperature/storage condition have been compared (Figure 3). Pearson correlation coefficient has been calculated for each relationship and was significant for each temperature/storage condition except for wet 15°C (it has been so discarded from all future regression line parameters comparison). Slopes and intercepts were tested with Analysis of variance in General Linear Model using storage period as covariate. The intercepts of the regression models are not significantly different (F = 0.75, DF = 7, P = 0.63) while slopes are significantly different (F = 14.67, DF = 7, P < 0.001). According to the Figure 3, the regression line of Bulk 20°C and Wet 20°C show the lower slope among all the regression lines (1.0 and 1.3 respectively) that also are not significantly different.

**Table 2:** **Effect of storage period, storage condition and temperature on percent hatch (± SD) of *An. arabiensis *eggs**

**Storage period (d)**	**Storage condition and temperature (°C)**								
	**Bulk**			**Dry**			**Wet**		
	**10**	**15**	**20**	**10**	**15**	**20**	**10**	**15**	**20**
1	78.6 ± 3.6	82.9 ± 1.2	83.3 ± 3.0	86.5 ± 1.9	87.0 ± 3.4	86.0 ± 2.8	82.1 ± 3.0	79.1 ± 0.3	81.6 ± 1.2
2	80.5 ± 2.6	78.0 ± 3.8	80.3 ± 2.6	81.6 ± 3.6	73.1 ± 4.8	75.9 ± 3.8	82.4 ± 2.8	76.7 ± 3.9	81.3 ± 0.9
3	56.0 ± 4.6	61.2 ± 7.0	78.6 ± 3.5	53.2 ± 7.7	52.6 ± 3.0	42.5 ± 8.0	56.6 ± 2.6	46.6 ± 10.2	69.1 ± 2.5
4	60.1 ± 4.7	56.2 ± 2.5	76.7 ± 2.8	49.9 ± 3.9	44.6 ± 5.1	39.8 ± 1.6	26.3 ± 3.5	60.6 ± 1.1	73.8 ± 3.9
8	33.1 ± 2.5	28.6 ± 4.4	75.6 ± 3.5	27.3 ± 2.5	22.3 ± 8.2	12.4 ± 1.2	25.3 ± 1.9	59.2 ± 0.8	72.1 ± 4.3

Based on these results, egg storage in bulk at 20°C was chosen as the appropriate environment for additional investigations on the effects of storage temperature and condition on life parameters such as larval development and adult emergence.

### Effects of egg storage duration on larval development parameters

Table 3 summarizes the results on the effects of egg storage duration in bulk condition at 20°C on larval developmental parameters. The developmental period from L1 to pupa and L1 to the adult stage did not differ significantly from the control. Surprisingly a shorter developmental time was observed for eggs stored at a longer duration. Percent survival from the L1 to the pupal stage was the same for up to 8 days of storage. Similar results were observed for survival up to the adult stage. The percent hatch for eggs up to 6 days of storage was about 77%, and not significantly different from the control. However, the hatch rate declined starting at 7 days of storage. In summary, no differences in larval development and the survival rate to adult emergence were observed when stored 1 to 8 days, and a significant decline in egg hatch was observed after 6 days of storage.

**Table 3:** **Effects of bulk storage on hatchability, larval duration, emergence and survival of *An. arabiensis *when eggs were stored at 20°C**

**Egg storage period (d)**	**Percentage hatching**		**Duration larval stages (d)**		**Duration L1 to adult emergence (d)**		**Survival L1 to pupal stage (%)**		**Survival L1 to adult emergence (%)**	
1	75.1 ± 2.3	b	10.2 ± 0.3	a	11.2 ± 0.3	a	95.3 ± 0.6	a	93.7 ± 0.8	a
2	82.8 ± 2.8	a	9.2 ± 0.3	bc	10.8 ± 0.2	a	94.5 ± 0.9	ab	93.0 ± 0.9	ab
3	78. ± 1.5	ab	9.8 ± 0.1	a	10.7 ± 0.2	ab	93.7 ± 0.0	ab	91.4 ± 0.9	ab
4	78. ± 2.4	ab	9.8 ± 0.4	a	10.8 ± 0.4	ab	93.0 ± 0.9	abc	91.4 ± 0.9	ab
5	79.9 ± 4.5	ab	9.3 ± 0.1	b	10.3 ± 0.1	bc	93.7 ± 0.8	ab	91.4 ± 0.9	ab
6	76.8 ± 4.5	ab	8.2 ± 0.2	bc	10.2 ± 0.2	cd	92.2 ± 0.6	bc	89.8 ±1.0	ab
7	68.9 ± 4.6	c	9.1 ±0.2	bc	10.2 ± 0.2	cd	93.0 ± 0.9	abc	89.8 ± 1.0	ab
8	68.9 ± 4.6	c	9.1 ±0.0	bc	10.1 ± 0.0	cd	93.0 ±0.5	abc	89.8 ± 0.96	ab
9	53.2 ± 6.0	d	8.8 ±0.1	c	9.8 ±0.2	d	90.6 ± 0.0	c	89.1 ± 0.6	b
11	22.6 ± 6.2	e	----		----		----		----	
Control	78.5 ± 4.8	ab	9.2 ± 0.6	bc	10.1 ± 0.6	cd	95.3 ± 0.6	a	91.4 ± 1.2	ab

For each parameter, different letters indicate significant differences between data within the same column.

### Volumetric estimation of dried eggs

**Figure 4:**
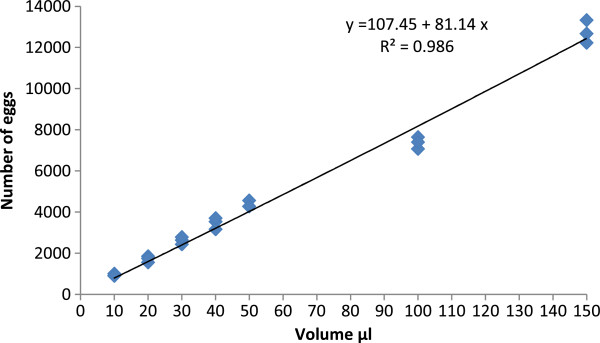
**Volumetric estimation of dried eggs of *An. arabiensis.***

Based on eggs counted from volumes between 10 and 150 μl, egg number and volume followed a linear relationship (y = 107.45 + 81.14x, *r* = 0.99 with *P* = 0.0001 and *R*^2^ = 0.986) (Figure 4). A total of 937, 1716, 2617, 3466 and 4370 eggs were counted from 10, 20, 30, 40 and 50 μl, respectively. The mean number of eggs in 150 μl was 12,734. There was a positive and significant correlation between the number of eggs and volume. The linear relationship was also examined using a simple linear regression equation. For each unit change in volume the number of eggs increased by 81. The goodness of fit test shows a very high value of *R*^2^ (*R*^2^ = 0.98), meaning that the model explains maximum variation caused by the regression coefficient.

## Discussion

Using a very simple apparatus with a wind speed of 1.8 m/s, approximately 40,000 eggs were dried in 20 min without any reduction in the percent hatch. When the size of the screen hole was increased from 6 cm to 9 cm, approximately the same number of eggs was dried in 10 min (I. Khan, personal observation). Tubergen *et al*[11] also used a simple apparatus to dry eggs of *An. stephensi* Liston, and stored dried eggs at 4°C. They reported an egg viability of 50% after five days in storage. In the present experiments more than 75% viability for up to eight days has been observed, and no significant reduction in larval survival was observed after the eggs were stored in bulk for eight days at 20°C.

The present results on egg viability after storage at various temperatures are not consistent with those reported by Bailey *et al.*[13] for *An. albimanus* eggs, where a 90% hatch rate was reported after storage of up to 7 days at 10–16°C. In this study, a 76% hatch after storage for up to 8 days at 20°C in bulk condition was recorded. A much lower hatch rate was observed after a storage duration of over 8 days at 10 or 15°C, (Table 2). Nevertheless, the findings are consistent with Bailey *et al*. for *An. albimanus* and *An. quadrimaculatus* on the high rate of egg viability when stored in bulk condition. In the present study, a 75–80% hatch rate in the control and after storage periods of up to 8 days has been observed. The normal hatch rate of *An. arabiensis* eggs is about 80%. Thus, evidently *An. arabiensis* eggs can be safely stored at 20°C for up to 6 days without any reduction in hatch rate, and for up to 9 days with no negative effects on the development and survival of the larvae. As the hatch rate of eggs following 11 days of storage in bulk was quite low, other parameters were not further studied.

A temperature lower than 20°C, permits storage for a few days but induces a decrease in the hatch rate with time; this suggests that a lower temperature has detrimental effects on *An. arabiensis* embryos. There are many studies on the influence of temperature at the early developmental stages of insects [17-22] and specifically those of mosquitoes including *Anopheles gambiae*[23], *Anopheles albitarsis, Anopheles aquasalis*[19], *Aedes aegypti*[24,25] and *Culex* spp. A strong influence of temperature on the embryonic development and hatchability of *An. albitarsis* and *An. aquasalis* has also been reported [26]. Microscopic observations on *An. gambiae* embryos showed that extreme low and high temperatures affected normal development [27]. This might be the case in the present study where a high rate of hatch and no significant hindrance in development was observed when eggs were stored in bulk at 20°C.

An accurate and simple method to estimate the quantity of dried eggs has been developed, allowing for the quantification of several thousand eggs. In mass rearing conditions, larval development in a large 100 (l) x 60 (w) x 3 cm (d) rearing tray resulted in the production of more than 3500 pupae from 4000 L1 larvae [20]. Knowing that the natural hatch rate of the Dongola strain after drying and storage is about 70%, 5700 dried eggs per tray are needed. With the equation established [volume of eggs = (number of eggs - 107.45)/81.14], the volume of eggs for 69 μl can be estimated. A little spoon with a volume of 69 μl could be created to allow for a rapid distribution of eggs into numerous trays from a stock of dried eggs collected from mass rearing cages.

## Conclusion

*Anopheles arabiensis* eggs can be stored in bulk condition at 20°C for up to 6 days without any reduction in hatch rate and up to 9 days with no significant negative impact on larval development.

## Competing interests

The authors declared that they have no competing interests.

## Authors’ contributions

The initial experimental set up was developed, executed and manuscript drafted by I. K. D. D., performed statistical analysis and helped in the development of the manuscript. S. M.S. helped in maintaining the laboratory colony, J.R.L.G., managed the overall experiment and critically reviewed the final draft of the manuscript. All authors approved the final version of the manuscript.
